# Surveying and Modelling 21st Century Online Learning Patterns of Medical Students

**DOI:** 10.3390/ijerph191912648

**Published:** 2022-10-03

**Authors:** Siya Liang, Ching Sing Chai, Vivian W. Y. Lee

**Affiliations:** 1 Centre for Learning Enhancement and Research (CLEAR), The Chinese University of Hong Kong, Hong Kong, China; 2 Faculty of Education, The Chinese University of Hong Kong, Hong Kong, China

**Keywords:** medical students, online medical education, online learning, 21st century learning models, learning competencies of 21st century

## Abstract

Medical education in the 21st century is shifting more toward online learning because of extensive application of information and communication technology (ICT). We surveyed medical students’ 21st century online learning experiences and modeled the interrelations among relevant dimensions of 21st century online learning. Based on the general themes proposed by multiple 21st century learning frameworks and current medical education emphases, a seven-factor instrument was developed for surveying 364 medical students’ learning process, thinking process, and basic science-related clinical ability. The associations among the seven factors and the structural relationships of how online learning practices and thinking processes affected basic science-related clinical ability were explored. The developed instrument was validated and possessed good reliability. The seven dimensions were interrelated. Specifically, meaningful learning with ICT was positively associated with other learning practices. The learning practices were positively associated with the thinking processes and the thinking processes were positively associated with students’ basic science-related clinical ability. Our findings suggested that students engaged in active and collaborative learning with technology would employ higher-order thinking and perceived better basic science-related clinical ability. The findings support engaging medical students with 21st century learning practices to strengthen students’ self-perception of clinical ability.

## 1. Introduction

A renewed focus of health care in the 21st century is required because of the changing environment [1]. The demands for renewal of medical knowledge and technological advancement trigger ongoing medical educational reforms to transform teacher-centric pedagogy. Over the past two decades, medical educators have embraced student-centered teaching modes with the use of information and communication technology (ICT) tools [2,3]. The shift has recently been accentuated towards extensive use of online or blended mode due to the COVID-19 pandemic. To cultivate students’ ability to adapt to the rapid changes, an educational model that foster 21st century learning practices emphasizing collaborative and intentional construction of knowledge supported by online environments has been advocated [4]. Thus, effective use of ICT (e.g., using online learning platform for advanced and latest knowledge acquisition and construction) has become an indispensable for the 21st century medical education. In the study of Stoehr et al., there is an apparent discrepancy between medical students’ preference and what actual curricula offer them [5]. The study indicates the necessity to investigate medical students’ online learning practices for curriculum reforms. Nevertheless, there remained an absence of instrument to survey medical students’ 21st century online learning.

### 1.1. Composition of the 21st Century Online Learning Survey (21 CLS) for Medical Students

Multiple 21st century learning models and reviews of future medical education have stressed the importance of teamwork, communication skills, use of ICT as tools for knowledge construction, self-directed learning competency, creative and critical thinking, problem-solving competency, and the ability to apply basic science to medical practice and diagnosis [3,6,7,8,9]. Nonetheless, rigorous survey among medical students in these aspects is lacking.

Sang et al. developed an instrument to evaluate 21st century learning competencies of teachers. The included subscales are collaborative learning (CoL), self-directed learning (SDL), meaningful learning with ICT (MLT), creative thinking (CreT), critical thinking (CriT), and authentic problem solving (APS). The proposed model is basically congruent with the focuses of 21st century medical education [10]. As the above components are key elements in understanding 21st century online learning [4], it could be adapted to measure medical students’ perception of online learning practices. Hence, we developed an instrument based on the study of Sang et al. to survey medical students’ 21st century online learning. However, specific items for assessing the professionalism of health-professional students are not included in the original 21st century learning survey (21 CLS).

Knowledge of basic science is a prerequisite for lifelong medical problem-solving, competent medical practice, and improvement of diagnostic accuracy [11,12]. There is a global consensus that competencies in basic medical sciences are essential in 21st century clinical medicine [8,9]. The ability to apply basic science to clinical practice is thus an important indicator of medical students’ ability-based educational outcomes in school [13]. Brown Medical School inaugurated a competency-based curriculum and developed the rules for students’ evaluation, which highlighted the roles of using basic science in the practice of medicine [14]. Hence, we adopted the ability (Using Basic Science in the Practice of Medicine) proposed by Brown Medical School and developed a subscale that aims to understand students’ self-efficacy of applying basic science into clinical practice (ABS) in the 21 CLS as one possible outcome variable when surveying medical students’ 21st century online learning.

### 1.2. Intercorrelations of the 21 CLS Components

The associations among various dimensions of the 21 CLS have been discussed. Sang et al. demonstrated strong intercorrelations among the six types of learning practices (*rs* > 0.50) [10], and the structural relations were re-verified in the later study [15]. However, the six learning practices present distinctive focuses, with MLT, CoL, SDL being considered as learning process denoting active learning within the socio-technological milieu, and CreT, CriT and APS being categorized as thinking process denoting higher-order cognitions. A plethora of studies have provided positive evidence on the impacts of learning process on thinking process enhancement. First, under online learning, use of ICT becomes the base of learning [16,17]. The employment of ICT enables the design of active and collaborative learning environments [18,19], and ICT as a learning medium can promote self-directed learning readiness [20,21]. Further, students engage in CoL that involve the active exchange of ideas would stimulate higher levels of CriT and CreT, which contribute to better problem-solving skills [22,23]. SDL plays a strategic role in planning, monitoring, and adjusting thinking and learning processes. It undergirds students’ cognitive functioning [24]. Going forward, problem solving involves complex thinking strategies [25], CriT and CreT may in turn predict APS. The six learning strategies are intimately associated, and previous literature suggest some predictive relations among them.

Promising pedagogical design of learning strategies in medical education (i.e., MLT, CoL, SDL, CriT, CreT, APS) that enrich students’ learning experiences in integrating, analyzing and applying scientific knowledge and information will equip students with intellectual capacity to use basic science knowledge and principles to clinical practice [26,27,28]. Thus, learning process and thinking process are likely to predict medical students’ ability in applying basic science into medical practice. The relations can be interpreted by the “presage–process–product” (3P) model proposed by Biggs [29], in which learning processes fuel the thinking processes and foster ability development. Nonetheless, the mechanism of how medical students’ practical ability can be enhanced through learning and thinking process have rarely been empirically investigated.

Medical students have been surveyed about their learning styles, such as learning approaches and orientations and conditions for performance [30]; however, little data exist on evaluating medical students’ 21st century online learning practices, especially in the period that the emergence of ICT and the outbreak of the COVID-19 pandemic accelerated the switch from face-to-face to online or blended learning. This sudden switch might have caused problems in both curriculum framework and students’ learning. Therefore, surveying medical students’ online learning experiences is necessary. Additionally, notwithstanding medical students’ practical ability has received increasing focus, there remained an absence in standardized tools for assessing medical students’ competence in applying basic science knowledge to clinic setting [31], thus it is of importance to take medical students’ ability in applying scientific knowledge to medial practice into consideration when designing the instrument. The proposed measurement can provide evidence to understand the important learning practices students are engaged in during online learning and throw light on curriculum design.

**Figure 1:**
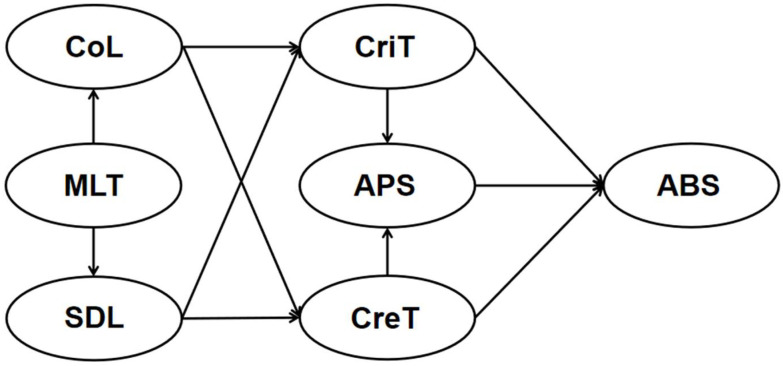
The hypothesized model for medical students’ 21st century online learning patterns.

To fill the research gap, this study aimed to (1) develop and validate a 21 CL instrument to survey and model medical students’ online learning practices; (2) explore the role of MLT plays in the development of other learning processes during online learning, and the mechanism of how learning process and thinking process influence the medical students’ basic science-related clinical ability. Particularly, we examined whether MLT serves as a base for the development of CoL and SDL, whether learning process (CoL and SDL) can predict thinking process (CreT and CriT), and whether CreT and CriT can affect APS and in turn lead to changes in ABS (Figure 1).

## 2. Materials and Methods

Data were collected from a 2021 to 2022 cohort. In view of the outbreak of COVID-19, all medical students were experiencing online learning during this period. Based on our investigation, Zoom was the primary tool for online teaching and learning, followed by Microsoft team and Blackboard. Medical students were provided with online lectures, watched patient videos and case scenarios, had group discussions, practiced medical training, submitted homework, and took examinations through the online learning platforms.

The Associate Director of the Faculty of Medicine acted as a gatekeeper to recruitment, forwarding emails of invitation, consent form as well as the survey link to all Year 1 to Year 6 medical students on behalf of the research team. All the data were collected through the university online platform. Participants were recruited through mass email to the students in the Faculty of Medicine, those with interest were asked to click into the link to the 21 CLS and complete the online survey. Informed consent was first obtained from the participants prior to their participation in the study.

### 2.1. Instrument

The 21st Century Learning Survey (21 CLS) developed by Sang et al. was adapted in the current study [10]. The original 21 CLS comprised of six subscales: CoL, CriT, SDL, CreT, MLT, and APS. Previous study has demonstrated good construct validity and internal consistency of the original 21 CLS. In our study, ABS was added as an important indicator to survey the influence of 21st century online learning on medical students’ clinical ability [14]. Thus, the developed 21 CLS is a seven-factor instrument that consists of 29 items, with each subscale containing 4 to 5 items, respectively. All items in the survey were screened and reviewed by three medical experts and two educational experts to establish face validity before actual implementation. Responses are provided on a 7-point Likert scale ranging from 1 (strongly disagree) to 7 (strongly agree).

### 2.2. Data Analysis

The 21 CLS for medical students was developed and validated with a mixed method that involved literature review, classical theory test, expert input, and confirmatory factor analysis (CFA). Though the original 21 CLS has been well validated, due to the change of the application context and the emergence of specified items for medical students, re-validation was needed. We subjected the newly developed 21 CLS for medical students to the CFA procedures with the use of Mplus 8.0 [32]. Standardized skewness and kurtosis of the twenty-nine items provided evidence that the 21 CLS meet the assumption of the normality (<|1.50|) [33]. Maximum likelihood estimation was adopted to compute the model fit statistics. Specifically, CFA on the ABS subscale was first conducted to test its construct validity. Then, additional CFA on the correlated seven-factor solution was performed to test the model. Multiple fit indices were accounted for assessing the model, in particular, item should have interpretable loading (>0.50) and with no problematic cross-loading (>0.20). Comparative fit index (CFI) and Tucker Lewis index (TLI) above 0.90 and the root mean square error of approximation (RMSEA) and the standardized root mean square residual (SRMR) below 0.08 represented acceptable fit [34,35,36,37]. Additionally, the composite validity (CR) and average variance extracted (AVE) recommended by Hair et al. were computed, with CR valued above 0.60 and AVE valued above 0.50 demonstrating convergent validity [38,39].

Further, reliability test, item-scale correlations, and intercorrelations were performed to test the internal consistencies of the 21 CLS subscales as well as the whole 21 CLS, with Cronbach’s alpha above 0.65, and significant item-scale correlations and significant intercorrelation among subscales indicating good internal consistency and content validity.

**Figure 2:**
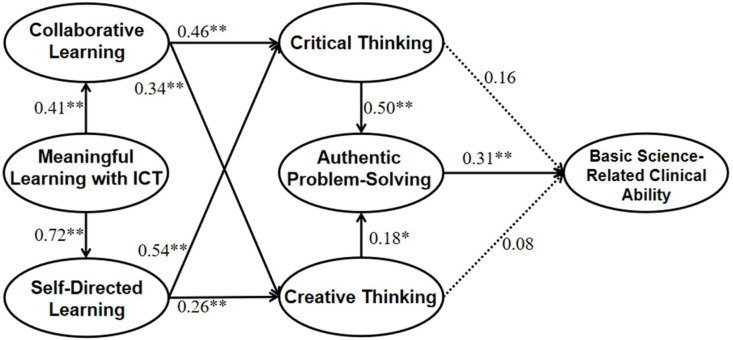
Path analysis with structural equation model on surveying medical students’ 21st century online learning patterns.Standardized coefficients for each path are presented in the figure. Dashed lines indicate non-significant paths. * *p* < 0.05; ** *p* < 0.01. N = 364.

Path analyses with structural equation model (SEM) was later adopted to explore the role of MLT plays in the development of other learning process (CoL, SDL), and the paths of how learning processes are related to the thinking processes (CriT, CreT), and how CriT and CreT predict APS, and further, how thinking processes predict ABS (Figure 2).

## 3. Results

### 3.1. Participants

A sample of 364 medical students (214 females, 58.8%) of different learning maturity from the Chinese University of Hong Kong were finally included in the study, with 214 of them (58.8%) from pre-clinical years (99 Year 1 students, 27.2%; 71 Year 2 students, 19.5%; 44 Year 3 students, 12.1%) and 150 of them (41.2%) from clinical years (77 Year 4 students, 21.2%; 52 Year 5 students, 14.3%; 21 Year 6 students, 5.8%). The age of the sample ranged from 18 to 30, specifically, with 189 of them between 18 and 20 years old, 158 of them between 21 and 23, 13 of them between 24 and 26, and the remaining 4 between 27 and 30.

### 3.2. Confirmatory Factor Analyses for the 21 CLS

CFA on the ABS yielded acceptable model fit, *χ*^2^ = 12.17, df = 5, *χ*^2^/df = 2.43, *p* < 0.05, RMSEA = 0.071, 90%CI [0.024, 0.118], CFI = 0.99, TLI = 0.98, SRMR = 0.018, suggesting that the ABS is a valid scale by itself.

CFA on the correlated seven-factor solution demonstrated good model fit statistics, *χ*^2^ = 619.97, df =356, *χ*^2^/df = 1.74, *p* < 0.001, RMSEA = 0.064, 90%CI [0.055, 0.072], CFI = 0.94, TLI = 0.94, SRMR = 0.053, indicating that the 21 CLS is a seven-factor model. Item loadings, composite reliability and average variance extracted for all the subscales are shown in Table 1. Overall, the fit indices indicated that the 21 CLS for medical students possess construct validity.

### 3.3. Agreement with 21 CLS for Medical Students, Subscale Intercorrelations and Internal Consistency

Table 1 summarizes the scale description and alpha coefficients for the 21 CLS for medical students. Alpha coefficients were above 0.90 for all subscales, and valued 0.95 for the total scale, indicating good internal consistency. Correlation analyses demonstrated strong internal consistency as well; specifically, each item correlated significantly with its corresponding subscale and items within the subscale (*ps* < 0.01), with CoL ranging from 0.72 to 0.91, CriT ranging from 0.66 to 0.91, SDL ranging from 0.73 to 0.91, CreT ranging from 0.78 to 0.95, MLT ranging from 0.52 to 0.91, APS ranging from 0.70 to 0.93, ABS ranging from 0.52 to 0.93. Agreement with each item and its corresponding subscale was obtained. Additionally, significant correlations were found among the subscales, ranging from 0.24 (MLT and CreT) to 0.68 (MLT and SDL), *ps* < 0.01 (see Table 2).

### 3.4. Structural Equation Model for Exploring the Patterns of Medical Students’ 21st Century Online Learning Practices

The proposed model in which MLT was entered as the base of the development of other learning practices (CoL, SDL), learning practices were entered as predictors to thinking processes (CriT, CreT, APS), and thinking processes were entered as predictors to ABS was computed, and the model yielded interpretable model fit, *χ*^2^ = 706.91, df = 366, *χ*^2^/df = 1.93, *p* < 0.001, RMSEA = 0.072, 90%CI [0.064, 0.079], CFI = 0.93, TLI = 0.92, SRMR = 0.077. Specifically, MLT significantly predicted CoL and SDL (MLT to CoL, *β* = 0.41, *p* < 0.01; MLT to SDL, *β* = 0.72, *p* < 0.01), CoL and SDL acted as important indicators to thinking process (CoL to CriT, *β* = 0.46, *p* < 0.01; CoL to CreT, *β* = 0.34, *p* < 0.01; SDL to CriT, *β* = 0.54, *p* < 0.01; SDL to CreT, *β* = 0.26, *p* < 0.01), and it was found that CriT and CreT could result in changes in APS (CriT to APS, *β* = 0.50, *p* < 0.01; CreT to APS, *β* = 0.18, *p* < 0.05), then APS further led to changes in ABS (APS to ABS, *β* = 0.31, *p* < 0.01; Figure 2).

## 4. Discussion

The objectives of the current study were to develop and validate the 21 CLS that can be adapted to medical students and to identify medical students’ 21st century online learning practices. Hence, the 7-factor solution 21 CLS with 29 items was constructed. The score agreement was acceptable to strong, revealing a good internal structure within each subscale. Reliability test and CFA on the whole scale and subscales indicated that the 21 CLS was reliable and valid. This study thus provides support for the 21 CLS being used as an instrument on understanding medical students’ 21st century online learning practices, thinking processes and ability-based medical outcomes. It could serve as foundation for other researchers who need to assess medical students’ online learning practices and to identify changes in any dimensions for evaluating the effectiveness of proposed 21st century online learning programs. Our findings also contribute to the literature on understanding the online learning practices of medical students from investigating the associations among learning process, thinking process, and learning outcome.

In line with previous literature, the correlations, internal coefficients, and interpretable fit statistics supported the 21 CLS constructs. The 21 CLS emphasizes 21st century medical online learning from the meaningful learning perspective with the use of ICT, collaborative teamwork, self-directed learning, critical and creative thinking skills, and problem-solving skills [3,4,7,40]. To ensure the online learning practices contributes to medical students’ clinical competences, assessment on basic knowledge of principles of different diseases and technologies in both classroom and community-based settings are considered in the instrument [8,11,41], thus ABS is added for measuring medical students’ ability in applying basic science knowledge to clinical practice.

When investigating the associations among varied aspects of medical students’ online learning experiences and the structural relationships for their medical educational outcomes, significant relations among learning practice, thinking process, and learning outcome were obtained. The finding expanded previous study indicating that MLT plays an essential role in the cultivation of CoL and SDL, which subsequently leads to changes in thinking process [15]. During online learning, student isolation and individual-centric modes of learning could impede students’ incorporating teamwork [22]. The emergence of ICT adds active elements to academic environment, providing opportunities for students to develop interpersonal skills, motivate and encourage the acquisition of knowledge and basic skills. ICT is thus considered as a powerful tool for enriching students’ educational experience [16,17]. The active learning environment incorporates elements of CoL and SDL that contributes to higher-order thinking processes could further support authentic problem solving [22,23,24]. The findings reveal that in this period when there were frequent switches between modes of instruction (online versus face to face), MLT is an important competence to facilitate medical students to engage in collaboration and self-direction, which in turns shape their thinking processes [23,42,43]. The structural model indicates that curriculum design for 21st century online medical learning needs to address all constructs measured as they are interrelated. As implied by Al-Kadri et al., the teacher plays a key role in initiating students’ learning process and determining what kind of learning strategies students adopt in clinical setting [43]. Medical educators will need to choose the ICT tools for specific authentic problems, structure SDL and COL, and provide scaffolding for the thinking processes. Future study may employ hierarchical modelling to investigate how medical teachers’ design of online learning shape students’ learning experiences.

Moreover, the improvement of APS would rely on the cultivation of one’s critical thinking and creative mind [44,45,46], which further influenced students’ basic science-related clinical ability (ABS) [8,9]. This finding is congruent with literature that thinking process can predict the change of ability-based medical educational outcomes [28]. Since the changes of thinking process are directed by learning process, the thinking process may act as a mediator in the link between learning process and basic science-related clinical ability. Future study may explore the mediating or moderating role of the thinking process by investigating the indirect effect of learning practices on ability-based educational outcomes through thinking process. The 21st century online learning experiences and the mechanism for change of thinking process and learning outcome of medical students in the current study showed similar patterns with previous literature [15] suggested that changes of educational outcomes could be driven by pedagogical strategies and thinking processes, and meaningful learning with ICT is essential under online learning [16,17]. However, the proposed associations of the online learning experiences of medical students in 21st century need to be further tested. Investigation would especially be meaningful if the teaching and learning practices have been intentionally designed to promote 21st century learning by medical educators, as suggested by Chai et al. [47].

As stated in the literature review, few studies have been targeted on identifying the online learning patterns of medical students in the 21st century. Thus, surveying medical students’ online learning experiences, thinking mode and skills has practical implications for assessing the learning environment students perceived and surfacing the discrepancies between the pedagogical design and actual implementation and outcomes. Furthermore, assessing students’ 21st century online learning could provide an insight on curriculum design and future educational policy making for medical students. In view of the rapid switch of online mode and the particularity of medical education, a deliberate curriculum framework for online learning remained absent in most of the medical schools [5]. A tailor-made learning design for students has been highlighted by Chacko in filling in the gap of the expectations from teacher and student [48]. When designing learning program and activities to improve 21st century online learning, various dimensions, including students’ learning maturity, needs in different stages, and their learning progress, should be taken into consideration to facilitate medical students to develop their professionalism. The results of path analysis also suggest that the associations of various dimensions can be considered in curriculum design for facilitating a specific dimension.

This study has several limitations. First, participants of the current study were voluntary-driven, thus the composition of the sample did not show equality regarding students of pre-clinical and clinical year and students of male and female genders. To extend the psychometric properties of the 21 CLS, future study could perhaps test measurement invariance on medical and health care students of pre-clinical and clinical year as well as students of different genders to examine if the proposed construct of the 21 CLS behaves equally among students with various demographic characteristics. Second, the data was collected from Hong Kong and may not be representative of other regions of different socioeconomic status. Third, the 21 CLS is a self-report survey that may have bias because of subjectivity. Future study can further test the validity of the instrument with a larger sample and different educational contexts and include measures such as in-depth interview and academic performance for surveying medical students’ 21st century learning patterns.

## 5. Conclusions

In conclusion, this study develops and validates an instrument for surveying and modeling medical students’ 21st century online learning patterns. Associations among various factors and their directions were explored, and our finding supported the hypothesized model and extended the previous literature.

## Figures and Tables

**Table 1:** Descriptions of the 21 CLS items, together with the Cronbach’s alphas, CR and AVE of latent variable.

Items	Mean	SD	Skew *	Kurt *	Item Loading
**Collaborative Learning and Professionalism (CoL)**	**Cronbach’s *α* = 0.92, CR = 0.92, AVE = 0.73**				
1. In this class, my classmates and I discuss different views we have and about things we are learning.	4.75	1.34	−0.53	−0.14	0.87
2. In this class, I get helpful comments about my work from other classmates.	4.61	1.36	−0.47	−0.15	0.83
3. In this class, my classmates and I actively work together to learn new things.	4.74	1.35	−0.51	−0.15	0.89
4. In this class, my classmates and I actively share and explain our understanding.	4.77	1.30	−0.50	−0.16	0.83
**Critical Thinking (CriT)**	**Cronbach’s *α* = 0.91, CR = 0.91, AVE = 0.73**				
1. In this class, I distinguish what is supported by evidence and what is not.	5.12	1.09	−0.53	0.67	0.76
2. In this class, I think about other possible ways of understanding what I am learning.	5.22	1.12	−0.68	0.90	0.88
3. In this class, I evaluate different opinions to see which one makes more sense.	5.21	1.13	−0.67	1.08	0.91
4. In this class, I decide what kind of information can be trusted.	5.15	1.09	−0.62	0.77	0.85
**Self-Directed Learning (SDL)**	**Cronbach’s *α* = 0.93, CR = 0.91, AVE = 0.71**				
1. In this class, I set goals for my studying.	5.47	1.24	−0.87	0.86	0.86
2. In this class, I check my progress when I study.	5.47	1.13	−0.82	1.29	0.85
3. In this class, I think about different ways or methods I can use to improve my study.	5.54	1.16	−0.85	1.18	0.82
4. In this class, I reflect on the ways I study.	5.53	1.12	−0.76	1.10	0.84
**Creative Thinking (CreT)**	**Cronbach’s *α* = 0.95, CR = 0.84, AVE = 0.95**				
1. In this class, I suggest new ways of doing things.	4.38	1.39	−0.24	−0.28	0.92
2. In this class, I design objects that may be helpful.	4.18	1.49	−0.25	−0.39	0.87
3. In this class, I produce ideas that are likely to be useful.	4.49	1.36	−0.40	0.05	0.94
4. In this class, I develop innovative ideas.	4.26	1.41	−0.24	−0.24	0.92
**Meaningful Learning with ICT (MLT)**	**Cronbach’s *α* = 0.90, CR = 0.95, AVE = 0.83**				
1. In this class, my classmates and I actively communicate online (e.g., LMS, Discussion Forum, Facebook, Wiki, WhatsApp, WeChat etc.) to learn new things together.	5.29	1.22	−0.74	0.60	0.65
2. In this class, I find out useful information on the Internet to facilitate my learning.	5.69	1.17	−0.83	0.45	0.89
3. In this class, I use the computer to organize, retrieve and save the information for my learning. (e.g., Blackboard.)	5.72	1.15	−0.75	0.40	0.88
4. In this class, I can use information technology to access accurate and reliable medical information.	5.68	1.12	−0.92	0.95	0.91
**Authentic Problem Solving (APS)**	**Cronbach’s *α* = 0.94, CR = 0.93, AVE = 0.77**				
1. In this class, I learn about real-life problems.	5.34	1.14	−0.74	1.06	0.89
2. In this class, I investigate the reasons that give rise to real-world problems.	5.28	1.17	−0.66	0.66	0.90
3. In this class, I apply the knowledge I have to solve real-life problems.	5.22	1.15	−0.49	0.52	0.89
4. In this class, I can simultaneously apply and integrate the medical knowledge I learn to diagnose a problem.	5.24	1.15	−0.53	0.52	0.82
**Ability of using Basic Science in the Practice of Medicine (ABS)**	**Cronbach’s *α* = 0.93, CR = 0.89, AVE = 0.63**				
1. I am able to describe the normal structure, function, and development of the human body.	5.22	1.01	−0.39	0.42	0.80
2. I can describe the risk factors, pathophysiologic mechanisms, structural and functional changes, and consequences of the underlying health problem.	5.02	1.14	−0.70	0.95	0.84
3. I can develop a therapeutic plan, incorporating risks and benefits, based on the mechanistic understanding of disease pathogenesis.	4.57	1.38	−0.59	0.02	0.87
4. I can measure and analyze the effectiveness of an applied intervention.	4.58	1.36	−0.56	0.14	0.88
5. I can articulate the pathophysiologic and pharmacologic rationales for the chosen therapy and expected outcomes at an appropriate level.	4.62	1.33	−0.61	0.31	0.52

* Abbreviation: Skew., Skewness; Kurt., Kurtosis.

**Table 2:** Correlation Matrix of the 21 CLS.

Factor	CoL	CriT	SDL	CreT	MLT	APS
CriT	0.54 **					
SDL	0.43 **	0.60 **				
CreT	0.49 **	0.54 **	0.38 **			
MLT	0.65 **	0.55 **	0.68 **	0.24 **		
APS	0.43 **	0.57 **	0.59 **	0.38 **	0.62 **	
ABS	0.29 **	0.44 **	0.39 **	0.40 **	0.28 **	0.44 **

** *p* < 0.01.

## Data Availability

Data available on request from the authors.
